# Self-Medication Practices Among the General Population in Al-Baha City, Saudi Arabia: A Cross-Sectional Study

**DOI:** 10.7759/cureus.50810

**Published:** 2023-12-19

**Authors:** Saja Mohammed S Alghamdi, Rayan Abdullah J Alzahrani, Sarah Saleh A Alghamdi, Rayan Murdhi A Alzahrani, Hanin Ayed A Alghamdi, Dalal Ayed M Alghamdi, Mohammed Ali S Alzahrani, Amr A Fouad, Rajab A Alzahrani, Mohammed A Alghamdi

**Affiliations:** 1 Medical School, Faculty of Medicine, Al-Baha University, Al-Baha, SAU; 2 Medical School, Al-Baha University, Al-Baha, SAU; 3 General Practice, King Fahad General Hospital, Al-Baha, SAU; 4 Department of Pharmacology and Therapeutics, Faculty of Medicine, Al-Baha University, Al-Baha, SAU; 5 Unit of Otolaryngology, Department of Surgery, Faculty of Medicine, Al-Baha University, Al-Baha, SAU

**Keywords:** adverse effects, prevalence, awareness, al-baha, self-medication

## Abstract

Background

The practice of self-medication (SM) is the use of self-consuming medication without consulting healthcare which carries its own risks. SM patterns differ across populations and are influenced by several factors. This study aimed to assess the prevalence of SM practices in Al-Baha, Saudi Arabia, to identify the factors contributing to this practice and develop effective strategies to decrease its occurrence and associated risks.

Methodology

This cross-sectional study was conducted in Al Baha Province, Saudi Arabia, over two weeks in July 2023, with a sample of 580 participants. Eligible participants were males and females, both Saudi and non-Saudi, aged 18-65. The data were collected using a self-administered online questionnaire.

Results

Of all participants, 48.7% admitted taking medications without a healthcare practitioner’s prescription in the last three months. Analgesics were the most common SM (29.1%), followed by vitamins and minerals (16.2%), and antipyretics (14.1%). The side effects experienced from SM included nausea (24.5%), headache (20.5%), and shortness of breath (8.7%). Regarding the source of medication, the majority (61.9%) obtained medications from a pharmacy and 14.6% used existing stock. Age was significantly associated with higher rates in the 18-29 and 40-49 age groups. Educational status was significantly associated with higher SM among graduates. Regarding reading medical instructions, 39.2% always read, 47.7% sometimes read, and 13.1% never read. Regarding antibiotic SM, 61 participants reported using over-the-counter (OTC) antibiotics. Common reasons for use included sore throat (27.8%) and common cold (19.6%). The most common reason for SM was to save time (25.9%), followed by avoiding crowds and long waits (17.1%).

Conclusions

A significant prevalence of SM practice concerning antibiotic misuse and sex differences with female dominance was detected. We recommend further public awareness activities from related organizations and more regulations for OTC prescription practices to ensure safe SM practices. In addition, further research is needed to explore SM patterns.

## Introduction

Self-medication (SM) involves the use of medication without consulting a healthcare provider or obtaining a prescription. This particularly concerns over-the-counter (OTC) medications, which the US Food and Drug Administration considers safe and effective for use without professional advice [1]. The appropriate use of non-prescription drugs to address health concerns entails responsible SM, whereas acquiring prescription medications without a doctor’s guidance is considered irresponsible SM [2,3]. Such practices contribute significantly to increased healthcare costs owing to adverse drug reactions and interactions, thereby imposing a substantial economic burden [4]. Common OTC medications include pain relievers, such as acetaminophen and ibuprofen [5]. Despite their easy accessibility, users must be cautious of potential risks such as improper dosing, drug interactions, and adverse effects [2].

SM patterns differ across regions and populations and are influenced by factors, including gender, age, education, self-care approach, medical history, illness severity, and socioeconomic aspects such as income, expenditure, and health insurance. These factors can result in medications obtained from sources such as home leftovers, pharmacies, or family members [3].

SM is more prevalent in developing countries and its incidence continues to increase worldwide [1,4]. This practice is more common in areas where purchasing prescription medications without prescriptions is facilitated and pharmacy practice laws and regulations are insufficiently enforced [4]. Improper SM can result in several potential hazards, including misdiagnosis, obscuring underlying symptoms, incorrect dosing, delayed professional medical care, adverse drug reactions, interactions with concurrent medications or food, drug abuse, and antimicrobial resistance. These problems can contribute to resource waste, excessive medication use, and superinfections among SM individuals [3,4]. Among the commonly used drugs in Saudi Arabia are painkillers, antibiotics, and proton pump inhibitors [6].

While several studies have reported the prevalence of SM among the general Saudi Arabian population [3,7], limited research has focused on specific regions, such as Al-Baha. Home to 339,174 residents, Al-Baha Province is one of the 13 provinces of Saudi Arabia, encompassing diverse environments such as Tihama, Badiyah, and As Sarrah.

This study aimed to investigate the prevalence of SM among the Al-Baha population and identify the factors contributing to this practice. By understanding the extent of SM in the region, we can develop effective strategies to decrease its occurrence and associated risks, ultimately improving the public health of Al-Baha residents.

## Materials and methods

Study design and setting

This cross-sectional study was conducted in Al-Baha Province, Saudi Arabia, over two weeks from July 10-25, 2023.

Participants

Eligible participants for the study were males and females, both Saudi and non-Saudi, aged 18-65 years, residing in the Al-Baha region. Participants were required to read and accurately complete a self-reported questionnaire. We excluded individuals who were not residents of Al-Baha, declined participation, or did not fully complete the questionnaire.

Data collection

Data were collected using a self-administered online questionnaire and disseminated to eligible individuals across the Al-Baha region. The questionnaire was hosted on Google Surveys, and the link was distributed by trained data collectors from different governorates in Al-Baha to ensure equal distribution. We primarily circulated the electronic survey through social media applications, most notably WhatsApp, to reach the target participants.

Variables

The survey covered the following items: (1) participant demographics (age, gender, education level, occupation, social status, and insurance status); (2) practices of SM, including conditions that led to SM, the medication used, reasons for SM, sources of information, sources of medication, expiration dates, and side effects; and (3) practices related to SM with antibiotics (condition, medication, and the dosage, duration, and source of information). Consent was obtained from the participants to proceed with the survey.

Data sources and measurement

The questionnaire used in this study was designed to draw insights from similar studies and surveys. The SM study questionnaire developed by Limaye was a significant reference for the development of our instrument [8]. The questionnaire was first drafted in English and then translated into Arabic to ensure comprehensibility among the local population. To confirm the accuracy and integrity of the translation, a back-translation process was performed.

To ensure content validity, the questionnaire was reviewed and refined using the inputs of two subject matter experts and a senior researcher. This step was crucial for enhancing the reliability of the data collection tool.

Before the final implementation of the questionnaire, we conducted a pretest using a small sample of 10 participants. This helped to spot and rectify any potential ambiguities in the questions or responses and ensure a smooth data collection process when the questionnaire was rolled out to the larger study population.

Sample size

The sample size was computed using the Raosoft sample size calculator [9], considering a 5% margin of error, 95% confidence level, a population size of 339,174, and a 50% response distribution. This resulted in a final sample size of 384. However, to minimize the sampling bias inherent to online studies, we aimed to collect responses from approximately 600 participants.

Ethical approval

This study was conducted in accordance with the principles of the Declaration of Helsinki. The study proposal was approved by the Institutional Review Board and Research Ethics Committee, Faculty of Medicine, Al-Baha University, Saudi Arabia (approval number: REC/PHA/BU-FM/2023/85).

Statistical analysis

Descriptive and inferential statistical analyses of the data were performed. Simple frequencies and percentages of the sociodemographic characteristics of the participants assessed for SM, sources, side effects, and other categorical variables were calculated and tabulated. The chi-square and Fisher’s exact test were used to determine the association between different sociodemographic features and SM. Statistical significance was established at p-values ≤0.05, with a 95% confidence interval. All statistical calculations were performed using SPSS version 29.0.0 (IBM Corp., Armonk, NY, USA).

## Results

Sociodemographic features of participants

The study included 761 participants, and 181 were excluded based on the exclusion criteria. Most participants were female (55.5%), married (61.6%), Saudi nationals (98.1%), and residing in Al-Baha (100%). The age distribution was diverse, with the majority aged 18-29 (36.9%). Regarding education, 60.2% graduated and 42.6% were employed. The monthly income varied, with 35.2% earning less than 5,000 SR, and 46.1% having no health insurance (Table 1).

**Table 1:** Sociodemographic features of participants assessed for self-medication.

		Frequency (n = 580)	Percent
Gender	Females	322	55.5
	Males	258	44.5
Age	18–29	214	36.9
	30–39	70	12.1
	40–49	175	30.2
	50–59	92	15.9
	>60	29	5.0
Nationality	Non-Saudi	11	1.9
	Saudi	569	98.1
Place of residence	Al-Baha	580	100
Marital status	Single	206	35.5
	Married	357	61.6
	Separate/Divorced	10	1.7
	Widow	7	1.2
Education	Up to secondary	21	3.6
	Higher secondary	111	19.1
	Graduation	349	60.2
	Postgraduation/PhD	50	8.6
	Diploma	49	8.4
Occupation	Employee	247	42.6
	House owner/Housewife	54	9.3
	Student	176	30.2
	Retired	65	11.2
	Business	15	2.6
	Other	23	4.0
Monthly income	<5,000 SAR	204	35.2
	5,000–10,000 SAR	118	20.3
	10,000–15,000 SAR	111	19.1
	15,000–20,000 SAR	100	17.2
	> 20,000 SAR	47	8.1
Kind of health insurance this year	Free care from a government hospital	256	44.1
	Private medical insurance	48	8.3
	No insurance	272	46.1

Perception and prevalence of SM among participants

Among 580 participants, 48.7% (287) admitted taking medications without a doctor’s prescription in the last three months. Regarding checking the expiry date, 27.5% checked it before use, 21.3% when purchasing, and 20.1% usually did not check. Medical instructions were always read by 39.2%, sometimes read by 47.7%, and never read by 13.1% of participants. Understanding medicine instructions was clear for 54.1%, partially incomprehensible for 44.7%, and incomprehensible for 13.1%. Regarding adverse effects, 58.4% stopped the medication, 13.5% went to the hospital, and 7.9% consulted a pharmacist (Table 2).

**Table 2:** Perception and prevalence of self-medication among participants.

		Frequency (n = 283)	Percent
Taken medications without a doctor’s prescription in the last 3 months	Yes	283	-
Check the expiry date	Usually forget	57	20.1
	When purchasing	58	21.3
	Before use	78	27.5
Read instructions before use	Always	111	39.2
	Sometimes	135	47.7
	Never	37	13.1
Understanding medicine instructions	Clearly	138	54.1
	Partially	114	44.7
	Incomprehensible	3	.5
What did you do during adverse effects	Stopped medication	52	58.4
	Went to the hospital	12	13.5
	Went to the pharmacist	7	7.9

Types of SM used and their adverse effects

**Figure 1:**
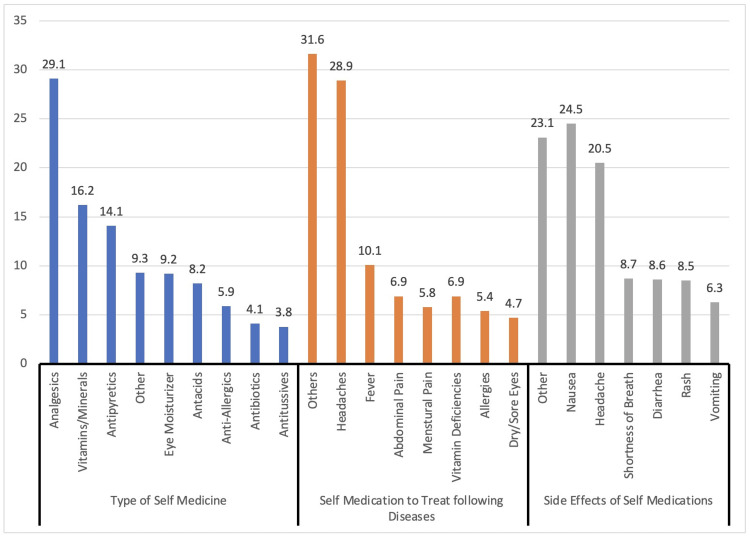
Types of self-medicines used to treat the diseases and their adverse effects.

The types of SM used to treat various diseases and their associated side effects are shown in Figure 1. Analgesics were the most common (29.1%), followed by vitamins and minerals (16.2%), and antipyretics (14.1%). Others included eye moisturizers, antacids, anti-allergens, antibiotics, and analgesics. Various diseases were treated with SM, with headaches being the most common (28.9%), followed by fever (10.1%), and abdominal pain (6.9%). Other conditions for which SM was used (31.6%) were diabetes mellitus, tooth pain, vomiting, diarrhea, constipation, and cough. The adverse effects experienced included nausea (24.5%), headache (20.5%), shortness of breath (8.7%), and others (23.1%) such as constipation, diarrhea, dry eye, drowsiness, and skin rash.

Source and motivation for SM

**Figure 2:**
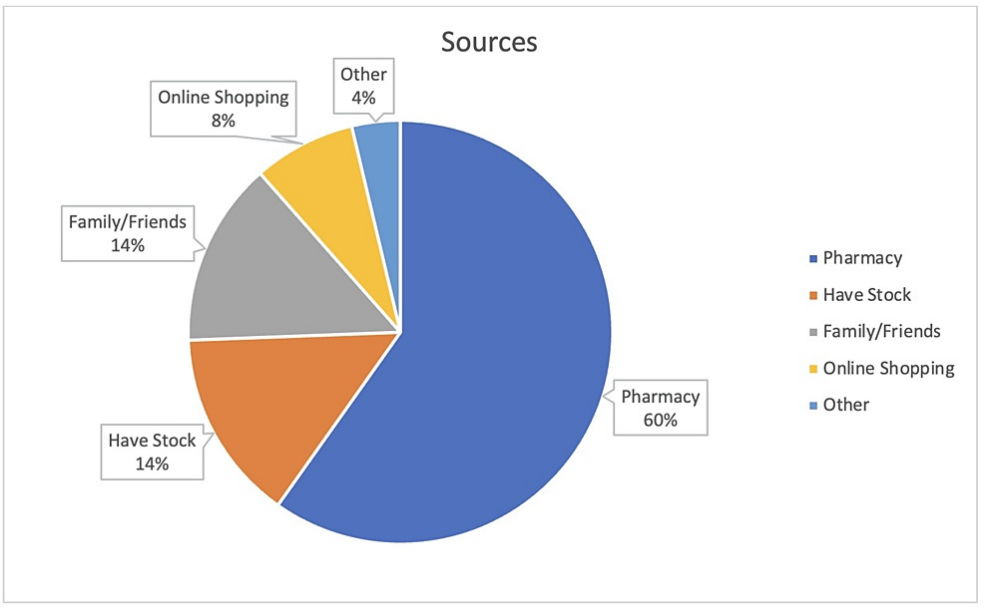
Different sources to obtain self-medications.

**Figure 3:**
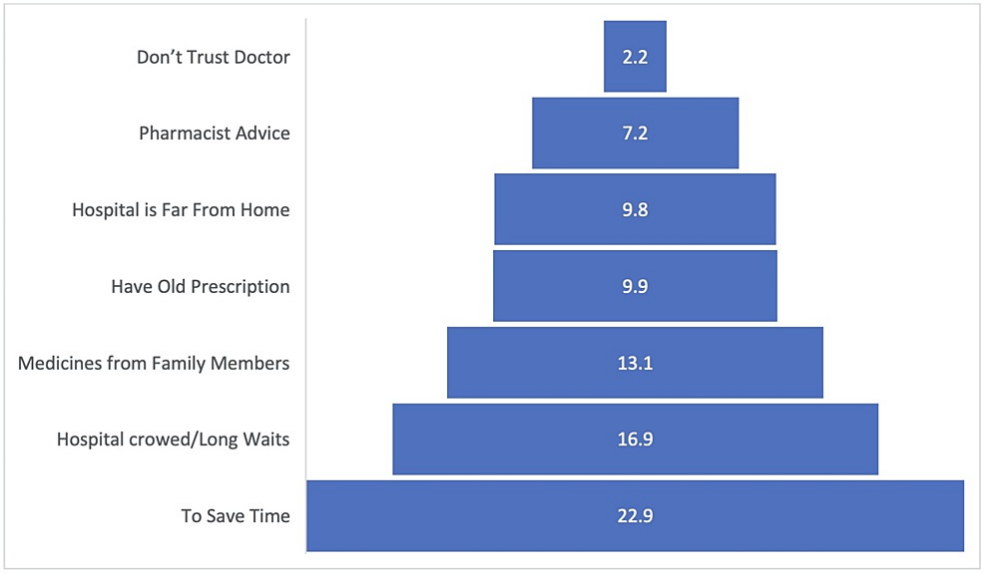
Motivations behind self-medications.

Figure 2 presents the sources from which participants obtained medicines for SM. The majority (61.9%) obtained their medicine from a pharmacy, 14.6% used existing stock, and 12.1% relied on family or friends to provide the medicine. Figure 3 shows the motivations behind SM among the participants. The most common reason was to save time (25.9%), followed by avoiding crowds and long waits (17.1%).

Perception and prevalence of antibiotics among participants

**Figure 4:**
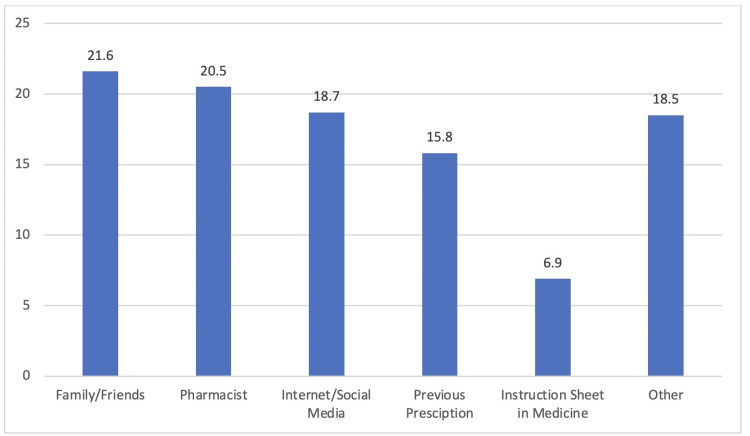
Different sources of information about antibiotic use.

Table 3 shows that 61 participants reported using OTC antibiotics. The common reasons included sore throat (27.8%), common cold (19.6%), and fever (18.1%). The amoxicillin-clavulanate combination was the most frequently used antibiotic (13.1%). Participants determined the dosage based on previous experience (34.4%) or pharmacist advice (22.9%). Some participants altered the doses (24.5%) and changed antibiotics without a prescription (24.5%). Antibiotics were discontinued after symptom relief (47.5%), course completion (27.9%), or after a few days (14.8%). The learning sources were pharmacists (27.8%) and previous experience (27.8%). Figure 4 shows that family and friends (21.6%), pharmacists (20.5%), and internet/social media (18.7%) were the key sources of information about antibiotic use.

**Table 3:** Perception and prevalence of antibiotics among participants.

		Frequency (n = 61)	Percent
Ever used over-the-counter antibiotics	Yes	61	-
Reason for antibiotic use	Fever	11	18.1
	Sore throat	17	27.8
	Common cold	12	19.6
	Infection	9	14.7
	Other	12	19.6
Most commonly used antibiotic	Amoxicillin-clavulanate	8	13.1
How do you know the dosage	Previous experience	21	34.4
	Consult a pharmacist	14	22.9
	By checking the medicine leaflet	14	22.9
	Internet	12	19.6
Change the dose of antibiotic	Yes	15	24.5
Major reason for dosage change	To prevent negative effects	13	26.2
Change the type of antibiotic	Yes	15	24.5
Reason for change	Cost	5	8.2
	Did not feel better with a previous type	6	9.8
	Reduce negative effects	6	9.8
When you stop the antibiotic	After few days	9	14.8
	After completing the course	17	27.9
	After the antibiotic is over	5	8.2
	After symptoms disappear	29	47.5
How do you know the course of treatment of the antibiotic	Pharmacist	5	27.8
	Previous experience	5	27.8
	Medication leaflets	5	27.8

Association between different factors and SM

Table 4 shows the associations between the different factors and SM among the participants. Gender was not significantly associated with SM (p = 0.393). However, age had a statistically significant association (p = 0.030), with higher rates in the 18-29 and 40-49 age groups. Nationality, residence, and marital status did not significantly influence SM use. Educational status was significantly associated (p = 0.003) with higher SM among graduates. The current occupation was also significantly associated (p = 0.010) with higher SM among both employees and students. Monthly income did not significantly affect SM use.

**Table 4:** Association of different factors with self-medication.

		Self-medications		Significance value
		No	Yes	
		N (%)	N (%)	
Gender	Female	170 (57.2)	152 (53.7)	0.393
	Male	127 (42.8)	131 (46.3)	
Age	18–29	104 (35.0)	110 (38.9)	0.030
	30–39	30 (10.1)	40 (14.1)	
	40–49	86 (29)	89 (31.4)	
	50–59	57 (19.2)	35 (12.4)	
	>60	20 (12.6)	9 (3.2)	
Nationality	Non-Saudi	7 (2.4)	4 (1.4)	0.405
	Saudi	290 (97.6)	279 (98.6)	
Marital status	Married	183 (61.6)	174 (61.5)	0.648
	Single	104 (35)	102 (36)	
	Separate/Divorced	7 (2.4)	3 (1.1)	
	Widowed	3 (1)	4 (1.4)	
Educational status	Up to secondary	15 (5.1)	6 (2.1)	0.003
	Higher secondary	64 (21.5)	47 (16.6)	
	Graduation	166 (55.9)	183 (64.7)	
	Postgraduation/PhD	19 (6.4)	31 (11)	
	Diploma	33 (11.1)	16 (5.7)	
Current occupation	Employee	117 (39.4)	130 (45.9)	0.010
	House owner/Housewife	32 (10.8)	22 (7.8)	
	Student	83 (27.9)	93 (32.9)	
	Retired	46 (15.5)	19 (6.7)	
	Business	6 (2.0)	9 (3.2)	
	Other	13 (4.4)	10 (3.5)	
Monthly income	<5,000 SAR	107 (36.0)	97 (34.3)	0.380
	5,000–10,000 SAR	67 (22.6)	51 (18)	
	10,000–15,000 SAR	49 (16.5)	62 (21.9)	
	15,000–20,000 SAR	52 (17.5)	48 (17.0)	
	>20,000 SAR	22 (7.4)	25 (8.8)	

## Discussion

In this study, total responses from 580 participants residing in Al-Baha were included in the data analysis. According to our findings, 48.7% of participants admitted to taking medications without a prescription. In contrast, other studies conducted in Qassim, Jeddah, and Mekkah, Saudi Arabia, revealed a higher prevalence (75.2 and 64.6%, respectively) [7,10]. Likewise, 60.7% of the participants used SM in the Eastern Province [3], and 49% were in the Central Province [2]. According to our data, over half of those surveyed were females (55.5%), indicating an association between female sex and SM. Another study revealed similar results to the current findings [1]. Similar results were reported in another study conducted in Hail, Saudi Arabia [11].

Regarding checking the expiry date, this study suggested that 27.5% checked it before use, 21.3% when purchasing, and the rest forgot to check. Overall, a likely explanation is the need to raise awareness regarding this issue as a portion of the store medicines at home can spoil over time. Hence, this is attributed to unfavorable adverse effects that could harm the patient if not checked for validity.

In the case of reading the medicine instructions, the present results showed that 39.2% always read it, whereas 13.1% never read it. A related study conducted in Eastern Province revealed that 33% of participants usually read the pamphlet [3]. On the other hand, in Central Province, 43.7% of participants always read the instructions, and 7.4% never read it [2].

Regarding adverse effects, 58.4% tended to stop the medication when experiencing side effects, whereas 13.5% went to the hospital. Our findings are similar to those of another study that revealed that 58.125% of cases showed adverse effects due to SM [3].

Based on participants’ responses, analgesics were the most commonly used SM in this study (29.1%). Other studies conducted in Najran, Jeddah, and Al Majmaah, Saudi Arabia, align with the findings of this research [4,8,12]. The widespread use of analgesics highlights the need to understand the reasons behind SM for pain management and ensure responsible usage. SM with vitamins and minerals (16.2%) and antipyretics (14.1%) in this work were lower compared to the studies mentioned above. The utilization of vitamins and minerals expresses an individual’s interest in maintaining their overall well-being, and antipyretics are frequently used to reduce fever, which is a common symptom of various diseases. The popularity of these SM choices suggests that individuals seek to address specific health concerns independently. The inclusion of eye moisturizers, antacids, anti-allergens, antibiotics, and analgesics among SM options highlights the diverse range of conditions that individuals attempt to manage on their own. This diversity reflects the complex nature of SM practices and the wide array of ailments which individuals seek relief from without professional medical guidance. Headaches were the most commonly addressed condition among diseases treated with SM (28.9%). Headaches are a common symptom experienced by many people, and the reachability of OTC pain relievers explains the high frequency of SM for headaches. Other studies conducted in Najran and Jeddah, Saudi Arabia, also showed that fever (10.1%) and abdominal pain (6.9%) were the most common reasons associated with SM practices [4,8].

In this study, the adverse effects reported by SM included nausea (24.5%), headache (20.5%), and shortness of breath (8.7%). The prevalence of adverse effects emphasizes the importance of understanding appropriate dosage, contraindications, and potential interactions when using SM options.

According to our findings, 61.9% of participants reported taking their medicine from a pharmacy, which is lower than the findings of another study conducted in Jeddah (89.6%) [8]. This indicates that pharmacies play an important role in facilitating SM. Pharmacies are accessible and offer a wide range of OTC medications, making them convenient choices for individuals. We also found that 14.6% of the participants relied on existing stocks. This means that some individuals prefer to keep a stock of medications at home. This tendency to rely on existing stock is attributed to previous experiences or the desire to have immediate access to medications without depending on external sources. Additionally, 12.1% reported dependence on family or friends to provide medicines for SM. This means that social networks and interpersonal relationships play crucial roles in SM practices.

Regarding the primary incentive for SM among the participants, the most commonly cited rationale was saving time (25.9%). This finding correlates with the convenience of SM. Another motivation for SM reported by 17.1% was avoiding hospital crowds and long waits. Factors such as limited healthcare resources, long waiting times, and the desire to avoid exposure to infectious diseases could contribute to this motivation.

The present results revealed that 61% reported using OTC antibiotics, which is higher compared to a study conducted in Riyadh, Saudi Arabia, which showed that 43% of participants used this kind of SM [13]. However, this is lower than the study done in Arar, Saudi Arabia (77.5%) [14]. The most common reasons reported in this study for antibiotic use were sore throat (27.8%), common cold (19.6%), and fever (18.1%). These findings imply that people tend to misuse antibiotics for viral infections, even though antibiotics are ineffective in such cases. Amoxicillin-clavulanate was the most frequently used antibiotic among the participants (13.1%), which is consistently common in studies conducted in Riyadh and Jeddah, Saudi Arabia [8,15]. This finding is explained by the widespread availability of these antibiotics in non-prescription settings. Therefore, it is important to raise awareness regarding the appropriate use of antibiotics and discourage the use of specific antibiotics without proper medical guidance to avoid potential adverse effects. Regarding dosage determination, a significant proportion of the study participants relied on previous experience (34.4%) and pharmacist advice (22.9%). This emphasizes the impact of personal experience and the influence of pharmacists in guiding antibiotic use. However, 24.5% of participants reported altering doses or changing antibiotics without a prescription. This inappropriate behavior can lead to insufficient treatment and antibiotic resistance.

Discontinuation of antibiotics after symptom relief was reported by 47.5% of participants, and 27.9% completed the full course. This sheds light on the importance of education on the competence of a full antibiotic course to reduce resistance. Moreover, 14.8% reported cessation of antibiotics after a few days, which further emphasizes the importance of educating individuals about the appropriate dosage and duration of antibiotic treatment. The information sources for antibiotic use were pharmacists (27.8%) and past experiences (27.8%). This suggests that participants depended on healthcare professionals and their previous encounters with antibiotics for guidance. Empowering pharmacists to provide accurate information and education about antibiotic use is vital for promoting responsible SM.

A previous investigation reported that individuals predominantly relied on their relatives, friends, and previous prescriptions as sources of information when attempting SM [16]. This study demonstrated relatively similar results, where the primary sources of information on SM with antibiotics were family/friends (21.6%), pharmacists (20.5%), internet/social media (18.7%), previous prescriptions (15.8%), and instruction sheets in medicine (6.9%). It is important to note that approximately 18.5% of participants had alternative sources of information. Further studies are necessary to pinpoint the exact sources that compel individuals to resort to antibiotic SM. However, relying on antibiotic-related information from unqualified sources can be risky and potentially harmful. Qualified sources can provide accurate and comprehensive information such as dosage, frequency, duration, contraindications, and interactions for a specific antibiotic [17].

A strong association between different factors and SM was reported, including age, sex, education, and income. A study conducted in the Middle East indicated a higher prevalence of SM among the middle-aged group (40-59 years), while others reported a higher prevalence among younger adults (20-39 years). The majority of studies demonstrated an elevated prevalence of SM among individuals with lower educational attainment and low or medium income [17-20]. This study showed higher rates of SM in two age groups (18-29 and 40-49 years), as well as among graduates, employees, and students. The analysis revealed that gender, nationality, residency, marital status, and monthly income did not exhibit any significant association with SM. In a similar study conducted in the Central Province of Saudi Arabia, a significant association between higher levels of education and SM was reported [2]. This could be due to higher access to information and resources, which potentially lead to SM. Overall, it is crucial for every person, regardless of their education level, to consult healthcare professionals and physicians for accurate diagnosis, treatment, and proper guidance on medication usage.

To our knowledge, this study on SM practice is unique as it is the first to be conducted among the general population in Al-Baha, Saudi Arabia. This study relied on self-reported data, which can be influenced by recall bias, as not all populations in Al-Baha were included. However, this is minimized by employing a well-structured, straightforward, and easily comprehensive questionnaire that was translated into the Arabic language, and data analysis was conducted on the total responses obtained from 580 participants.

## Conclusions

The present results revealed a significant prevalence of SM practice among the participants and gender differences in SM practices with female dominance. Variations in population awareness levels in understanding usage instructions demonstrate the need for more educational activities for the population.

Eventually, healthcare providers and physicians play a crucial role in providing proper education. Additional educational efforts are required to promote public awareness and encourage changes in this behavior. Implanting a strict set of guidelines on non-prescribed medications could potentially contribute to the attainment of this target. We recommend further public awareness activities from related organizations and more regulations on OTC prescription guidelines to ensure safe SM practices. We also recommend future studies, such as a systematic review and meta-analysis, examine the prevalence, practices, and factors associated with SM in Saudi Arabia.

However, limitations of this study include the reliance on self-reported data, which may be subject to recall bias. Additionally, more in-depth investigations regarding cultural and regional variations that might influence SM practices are required.
